# Autochthonous *Onchocerca lupi* infection of a domestic dog in Austria

**DOI:** 10.1186/s13071-023-05681-9

**Published:** 2023-02-01

**Authors:** Maria Sophia Unterköfler, Alexandra Huck, Katja Silbermayr, Hans-Peter Fuehrer

**Affiliations:** 1 Institute of Parasitology, Department of Pathobiology, University of Veterinary Medicine Vienna, Vienna, Austria; 2 Small Animal Practice Dr. Alexandra Huck, Ziegelgasse 20, Güttenbach, 7536 Güssing, Austria; 3 Boehringer Ingelheim RCV GmbH & Co KG, Dr. Boehringer Gasse 5-11, 1121 Vienna, Austria

**Keywords:** Canine onchocercosis, *Cytochrome c oxidase subunit I *gene (*COI*), Ocular helminthosis, PCR, Zoonotic

## Abstract

*Onchocerca lupi* is an emerging canine ocular pathogen with zoonotic potential. In Europe, known endemic areas are the Iberian Peninsula and Greece, but the parasite has also been found in Romania, Hungary, and Germany. A 5-year-old Irish Wolfhound was presented in August 2021 with ocular discharge. A subconjunctival granulomatous nodule containing several nematode fragments was removed. Molecular analysis of the mitochondrial *cytochrome c oxidase subunit I* gene confirmed the presence of *O. lupi* genotype 1. This is the first report of autochthonous *O. lupi* infection in a dog from Austria.

**Graphical Abstract:**

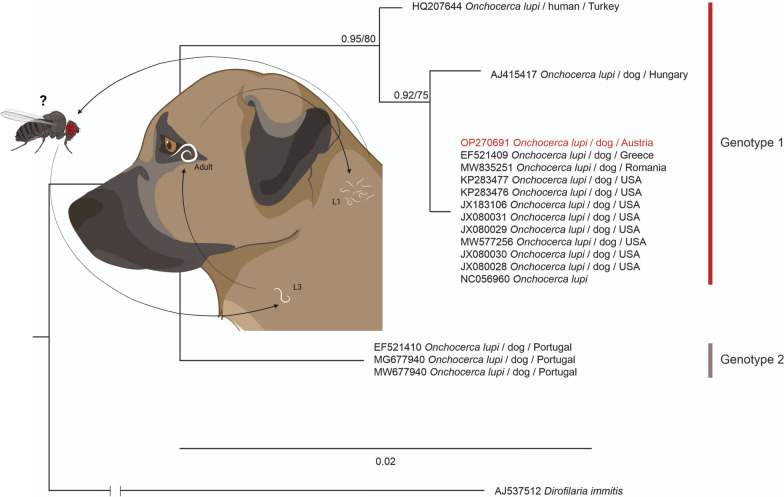

## Background

Species of the family Onchocercidae parasitize many different vertebrate hosts and include pathogens relevant to human health such as *Onchocerca volvulus*, the causative agent of river blindness [1]. *Onchocerca lupi* was first described in a wolf (*Canis lupus*) from Russia in 1967 and affects dogs (*Canis lupus familiaris*) and, to a lesser degree, cats (*Felis silvestris catus*). Moreover, humans can be infected as well [2–5]. The adult worm is most frequently found in the subconjunctival or subcutaneous tissue, but in humans spinal cord infections have been also reported [5–7]. Clinical signs may vary, and animals that present no obvious clinical signs may not be diagnosed for several years [8]. Based on the vector capacity of other *Onchocerca* spp. and the findings of *O. lupi* DNA in Simuliidae, these have been suggested as potential vectors [9]. Other arthropods have also been considered, but evidence of competent transmission is still missing [10–12]. This parasite has been documented in Europe, America, Africa, and Asia [1, 13–15]. In Europe, the Iberian Peninsula and Greece are known to be endemic areas, but cases have also been reported from Romania, Hungary, and Germany [8, 16–19]. Diagnosis can be based on adult specimen identification in clinical cases or by skin snips and detection of microfilariae [17, 20, 21]. Morphological identification can be confirmed by PCR of, for example, the mitochondrial *cytochrome c oxidase subunit I* (*COI*) gene [22]. Treatment recommendations include surgical removal of the parasite and the use of drugs such as macrocyclic lactones [1, 23]. The present report describes the first autochthonous *O. lupi* infection in Austria.

## Methods

In August 2021 a 5-year-old Irish Wolfhound living in Güssing district (Burgenland), which was born in Austria and had never left the country, was presented with ocular discharge. No other clinical signs were noted at physical examination. Subconjunctival granulomatous nodules containing nematodes were detected in both eyes and removed with forceps. The nodules were placed in saline solution and sent to the University of Veterinary Medicine, Vienna, where it was stored at −20 °C until further analysis. Nematodes were examined morphologically under a stereomicroscope, and DNA was extracted from fragments using a commercial DNA extraction kit (DNeasy^®^ Blood & Tissue Kit; QIAGEN, Hilden, Germany) according to the manufacturer's instructions. To obtain a fragment of the *COI* gene with 649 nucleotide positions, PCR was done on a fragment of one nematode using primers COIintF/COIintR [24] with the following amplifying temperature profile: initial denaturation at 95 °C for 2 min, followed by 35 cycles of 95 °C, 50 °C, and 72 °C each for 1 min, and final extension at 72 °C for 7 min. PCR products were run on 2% agarose gels stained with Midori Green. The PCR product was further analysed by Sanger sequencing (LGC Genomics, Berlin, Germany). The sequence was compared to available sequences using the BOLD and GenBank nucleotide basic local alignment search tool.

For phylogenetic analysis, nucleotide sequences of *O. lupi* available on the NCBI GenBank database were searched by using the BLAST function, using the sequence obtained in this study. The sequences were aligned and sorted using the default option (FFT–NS–2) in MAFFT v.7.311 [25], and sequences not covering the fragment of the sequences obtained in this study were excluded. Maximum likelihood (ML) and Bayesian inference (BI) trees were calculated based on the alignment, including 17 sequences (649 nucleotide positions). Sequences were collapsed to haplotypes using DAMBE v.7.0.5.1 [26], leaving four haplotypes. As outgroup, a sequence of *Dirofilaria immitis* (GenBank accession number: AJ537512) was used. ML bootstrap consensus trees (1000 replicates) were calculated using the W-IQ-TREE web server (http://iqtree.cibiv.univie.ac.at/; [27]) applying the model TIM3 + F + G4, which were suggested as best fit for the data set in the model test according to the corrected Akaike information criterion. The BI trees were calculated using MrBayes v.3.2.7 [28], applying the next complex model GTR + G, because the same model was not available in this program. The analysis was run for 10^6^ generations (number of chains: 4), sampling every 1000th tree. The first 25% of trees were discarded as burn-in, and a 50% majority-rule consensus tree was calculated based on the remaining 7500 trees.

## Results and discussion

**Fig. 1:**
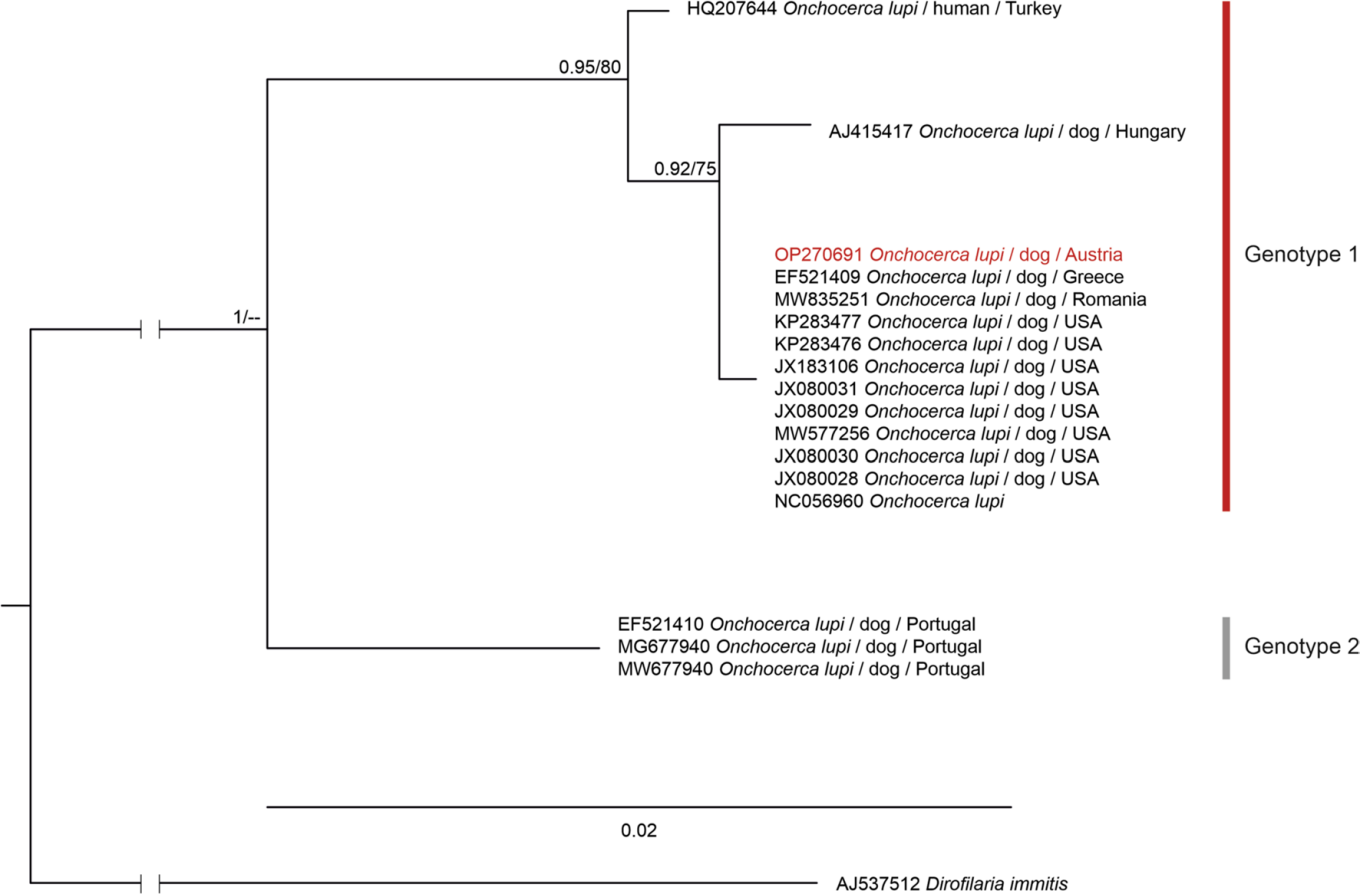
Bayesian interference (BI) tree featuring mitochondrial *cytochrome c oxidase subunit I* (*COI*) sequences (649 nucleotide positions) of *Onchocerca lupi*. Nodes are marked with BI posterior probabilities and ML bootstrap values. The sequence marked in red was obtained in the present study. Scale bar indicates the expected mean number of substitutions per site according to the model of sequence evolution applied

The dog was treated twice at an interval of 2 weeks with a combination compound containing moxidectin (2.5 mg/kg BW) and imidacloprid (10 mg/kg BW) Spot-On (Advocate^®^; Bayer AG, Leverkusen, Germany) for the control of *O. lupi* and with a topical ointment containing tobramycin and dexamethasone (Tobradex^®^, Novartis AG, Basel, Switzerland) to promote the healing of the eye inflammation. The clinical signs disappeared and did not recur within the follow-up period of 1 year. Treatment with moxidectin has been reported to be successful [23]. However, whether medical treatment or the surgical removal alone resolved clinical signs cannot be concluded with certainty. In addition, it is not clear whether the treatment eliminated all nematodes as no skin biopsies could be obtained before and after treatment because of the lack of owner consent. Morphological examination of the nodule revealed several worm fragments. The DNA sequence obtained was 100% identical to an *O. lupi* sequence documented in a dog from Greece (GenBank accession number: EF521409) and has been uploaded to BoldSystems^®^ (Process ID: PAVEA164-22) and GenBank (accession number: OP270691). This haplotype has been referred to as genotype 1 (Fig. 1), which occurs in northern America, southwestern Asia, and Europe, with the exception of the Iberian Peninsula, where genotype 2 is present [15].

In total, 45 species of Simuliidae, which could potentially act as vectors, are known to exist in Austria [29, 30]. In the Lafnitz River near Heiligenkreuz town (Burgenland), located near Güssing (Burgenland), *Simulium erythrocephalum, Simulium ibariense*, and *Simulium ornatum* have been found [29].

Coyotes (*Canis latrans*) have been considered as reservoir hosts in America [31]. In Europe, coyotes are not present, but other wild canids could probably fulfil this role. Another more likely mode of introduction is through pets travelling from endemic regions and subsequent establishment of the parasite in areas where it has not been present before [32]. To determine the current prevalence of *O. lupi* in Austria, a prevalence study should be performed in dogs and/or wild canids using skin snips and/or serology [8, 17, 33].

## Conclusion

Information on the treatment but also on transmission and distribution of this parasite is still scarce. This case report highlights that *O. lupi* can also be present in countries not yet classified as endemic and underlines the need to raise awareness of this zoonotic parasite.

## Data Availability

Additional data can be provided on request.

## Abbreviations

BI: Bayesian inference
*COI*: Mitochondrial *cytochrome c oxidase subunit I*
ML: Maximum likelihood
